# Reproductive Seasonality Affects In Vitro Embryo Production Outcomes in Adult Goats

**DOI:** 10.3390/ani11030873

**Published:** 2021-03-18

**Authors:** Joanna M.G. Souza-Fabjan, Lucas F.L. Correia, Ribrio I.T.P. Batista, Yann Locatelli, Vicente J.F. Freitas, Pascal Mermillod

**Affiliations:** 1 Faculdade de Veterinária, Universidade Federal Fluminense, Niterói, RJ 24230-340, Brazil; lucascorreia@id.uff.br (L.F.L.C.); ribrio@yahoo.com.br (R.I.T.P.B.); 2 Museum National d’Histoire Naturelle, Réserve Zoologique de la Haute Touche, 36290 Obterre, France; yann.locatelli@mnhn.fr; 3 Laboratório de Fisiologia e Controle da Reprodução, Universidade Estadual do Ceará, Fortaleza, CE 60714-903, Brazil; vicente.freitas@uece.br; 4 Institut National de Recherche Pour L’agriculture, L’alimentation et L’environnement (INRAE), Physiologie de la Reproduction et des Comportements, 37380 Nouzilly, France; pascal.mermillod@inrae.fr

**Keywords:** caprine, COC, IVF, IVP, IVEP, oocyte competence, photoperiod, reproductive efficiency, season, seasonal breeder

## Abstract

**Simple Summary:**

Reproductive seasonality is usually determined by photoperiod and may also be influenced by nutritional sources. Little is known about the effect of season on the efficiency of assisted reproductive technologies, such as in vitro embryo production in seasonal species. This study was conducted to generate an understanding of the seasonality influence on in vitro embryo production outcomes in goats. Overall, the breeding season improved oocyte developmental competence, with higher cleavage and blastocyst yield, while there was no difference in embryo quality throughout the years.

**Abstract:**

Reproductive seasonality may have a considerable influence on the efficiency of assisted reproductive technologies in seasonal species. This study evaluated the effect of season on cleavage, blastocyst rates and quality of in vitro produced (IVP) goat embryos. In total, 2348 cumulus–oocyte complexes (COCs) were recovered from slaughterhouse ovaries and subjected to the same IVP system throughout 1.5 years (49 replicates). The odds ratio (OR) among seasons was calculated from values of cleavage and blastocyst rates in each season. Cleavage rate was lower (*p* < 0.05) in spring (anestrus), in comparison with either autumn (peak of breeding season) or summer, while the winter had intermediate values. Furthermore, lower OR of cleavage was observed in spring. Blastocyst formation rate (from initial number of COCs) was higher (*p* < 0.05) in autumn (52 ± 2.5%) when compared with the other seasons (combined rates: 40 ± 1.9%). Moreover, its OR was higher (*p* < 0.05) in autumn compared to all other seasons and impaired in the spring compared to winter (OR: 0.54) and summer (OR: 0.48). Embryo hatchability and blastocyst cell number were similar (*p* > 0.05) among seasons. In conclusion, the breeding season leads to improved oocyte developmental competence, resulting in higher cleavage and blastocyst yield, whereas embryo quality remained similar throughout the years.

## 1. Introduction

It is well known that although some species have adopted strategies to breed year-round, others exhibit reproductive seasonality, breeding/calving only at a specific period of the year [1]. Reproductive seasonality is generally driven by photoperiod and may also be influenced by nutritional sources availability. In low latitude regions, day length remains steady throughout the year, but reproductive activities variations can be associated mainly to climatic conditions such as rainfall, impacting feed availability and quality [2]. However, in well-nourished mammals, photoperiod is the strongest reason for reproductive seasonality [3]. This is an adaptation that ensures offspring survival by calving at the period of higher roughage accessibility and nutritional quality [4]. When photoperiod changes from longer to shorter daylight, with more dark hours in a day, the photo receptors of the animal eye send this information to the pineal gland, increasing melatonin secretion, and goats restart their regular estrous cycles [1].

In general, in the northern hemisphere, the transition season in goats occurs in summer (June to September), the peak of breeding season is in the autumn (September to December), in winter (December to March) the occurrence of regular estrus relies more on the breed and animal intrinsic characteristics, while the deepest anestrus is detected in spring (March to June) (Reviewed by Chemineau, et al. [5]). In seasonal breeders, the reproductive seasonality may have a substantial impact on the efficiency of overall assisted reproductive technologies (ARTs, [4]). Considering the strong influence of seasons on small ruminants reproductive function due to hormonal alterations [6], it would be logical to suppose they may also affect the overall success of ARTs, such as in vitro embryo production (IVP).

In bovine, a nonseasonal species, the cleavage and morulae development rates were lower in the autumn compared with all other three seasons, while the blastocyst rate was the least when oocytes were collected during the summer season, probably due to hot weather and lower feed quality in IVP systems [7]. Curiously, the environmental temperature significantly affected women’s pregnancy rates after in vitro fertilization (IVF) in a sub-tropical region [8]. In studies assessing the effect of temperature, better results were reported in the cold season in buffalo [9] and in sheep [10], both being seasonal breeders.

In seasonal breeders, most research groups have reported consistent variations in embryo yield throughout the year. For instance, a higher number of oocytes presenting higher quality were recovered from Zandi ewes in the breeding season, allowing them to achieve higher blastocyst development [11]. Overall, higher IVP rates are obtained in the breeding season, compared to anestrus [11,12,13,14,15]. However, in prepubertal goats, after IVF, both cleavage and blastocyst rates were significantly higher in the nonbreeding season, compared to the breeding season (41° N latitude; [16]). It is not clear why these results are different to all others presented before; the reason for such disparity could be species-specific or an effect of the goat category/parity (prepubertal animals).

According to our current knowledge, the seasonal effects on embryo IVP have not been studied in adult goats. Regardless of the season, there are many factors that can directly affect the success of IVP, such as: oocyte origin, either collected from slaughterhouse ovaries or by laparoscopic ovum pick up [17], cumulus–oocyte complexes (COCs) recovery method [11], oocyte grading and selection [18], which is at least in part subjective, and the IVP conditions in the successive steps [17,19,20]. It is reasonable to suggest that all these factors must be controlled in order to specifically assess any season effect throughout the year. Therefore, this study was designed to investigate the effects of reproductive seasonality in adult goats on abattoir-derived oocytes’ developmental competence, over a period of 1.5 years.

## 2. Materials and Methods

All the chemicals were purchased from Sigma-Aldrich Chemicals Co. (St. Louis, MO, USA) except where otherwise specified.

### 2.1. Experimental Design

The experiment was performed during the four seasons: spring (nonbreeding season), summer (transition season), autumn (breeding season) and winter (end of breeding season, start of anestrus season) at the Reproductive Physiology and Behaviors Research Unit (PRC) in Nouzilly, France (latitude 47°22′ N, longitude 00°41′ E). Seasons were defined based on periods for equinoxes and solstices in the northern hemisphere. A total of 49 replicates (autumn: 17, spring: 7, summer: 15 and, winter: 10) of goat IVP were performed, using a total of 2348 COCs (autumn: 811, spring: 404, summer: 639 and, winter: 494). Two straws per replicate of semen from the same ejaculate/buck were used for six months and two straws (same ejaculate) per replicate of semen from another buck were used for 12 months. Data for embryo development were assessed over the period of 1.5 years. Several factors that could certainly affect the IVP outcomes were controlled in this study: only slaughterhouse-derived oocytes were used, the same COC recovery method was applied, the IVP protocol and system conditions were the same and all the steps were conducted by the same technician throughout the entire time.

### 2.2. Oocyte Recovery

Ovaries from adult dairy goats, regardless the stage of estrous cycle, were collected from a local slaughterhouse and transported to the laboratory in a thermal container with 0.9% NaCl solution at 32 °C within 3–4 h after collection. Ovaries were washed in pre-warmed fresh saline (32 °C), and COCs were aspirated from all follicles between 2 and 6 mm in diameter with an 18-g short bevel needle connected to a conic tube under controlled vacuum (30 mm Hg). The collection tube was previously filled with ⁓ 3 mL of HEPES buffered tissue culture medium 199 (TCM 199, M7528), supplemented with 20 µg/mL heparin (Choay, Glaxo Wellcome Production, Notre Dame de Bondeville, France), 25 µg/mL gentamycin (G1272) and 0.4 mg/mL fraction V bovine serum albumin (BSA; A9647) [21].

### 2.3. In Vitro Maturation (IVM) of Oocytes

The COCs were recovered under a stereo microscope (Nikon Corporation, Japan) and graded regarding their quality. Only good quality (Grade 1 and 2), i.e., surrounded by at least one complete layer of unexpanded cumulus cells and homogenous cytoplasm, were used for IVM [18]. All COCs were washed four times and transferred into 4-well plates (Nunc, Roskilde, Denmark), containing 40 to 50 oocytes in 500 µL of maturation medium. The maturation medium contained bicarbonate buffered TCM 199 (M4530) supplemented with 10 ng/mL epidermal growth factor (EGF; E4269) and 100 µM cysteamine (M9768). The COCs were incubated for 22 h at 38.8 °C in a humidified atmosphere of 5% CO_2_ in air [21].

### 2.4. Sperm Preparation and IVF of Oocytes

Sperm from frozen/thawed semen were centrifuged (15 min at 700 g) on 2 mL of Percoll (Pharmacia, Uppsala, Sweden) discontinuous (45/90%) density gradient. Viable sperm pellet was diluted in the adequate volume of fertilization medium to achieve a final concentration of 2 × 10^6^ sperm/mL, Day 0 being considered as the day of IVF [21].

The matured COCs were transferred into 35 mm Petri dishes and washed in fertilization medium, which consisted of synthetic oviduct fluid (SOF) medium (pH = 7.3, 280 mOsm), supplemented with 10% of heat-inactivated estrus sheep serum, 5 µg/mL heparin (Calbiochem 375 095) and 40 µg/mL gentamycin (G1272). Groups of 40 to 50 oocytes were transferred into 4-well plates containing 450 µL of fertilization medium and, after sperm preparation, 50 µL of sperm suspension were added to each well. Sperm and oocytes were co-incubated for 16–18 h at 38.8 °C in a humidified atmosphere of 5% CO_2_ in air [21].

### 2.5. In Vitro Development (IVD) and Embryo Quality

At the end of IVF all presumptive zygotes were placed into 15 mL conic tubes containing 2 mL of TCM 199 medium and 0.4 mg/mL BSA, and vortexed for 2 min (moderate speed) to remove cumulus cells. All presumptive zygotes were recovered in 35 mm plates, washed four times in culture medium (SOF supplemented with 3 mg/mL BSA) to remove spermatozoa and transferred by groups of 20–25 into 4-well plates containing 20–25 µL drops of culture medium covered with 700 µL of mineral oil (M8410). The presumptive zygotes were cultured for eight days at 38.8 °C in a humidified atmosphere of 5% O_2_, 5% CO_2_ and 90% N_2_. At Day 2, 10% fetal calf serum (MP Biomedicals, 2916748) was added directly into the culture droplets [21].

Embryos were examined morphologically under a stereomicroscope, and the efficiency of development was assessed as the percentage of cleaved embryos on Day 2, and the percentage of blastocysts on Day 8, expressed either over the initial number of oocytes subjected to IVM or the number of cleaved embryos at Day 2. On Day 8, samples of expanded blastocysts were washed to remove the mineral oil, fixed in ethanol and stained with Hoechst 33258 to count their total number of blastomeres. Cell counting was conducted under an epifluorescence inverted microscope (Diaphot, Nikon, Japan).

### 2.6. Statistical Analysis

The results of cleavage, blastocyst, and hatching rates were tested for normality by the Shapiro–Wilk test, before being subjected to analysis of variance (ANOVA), followed by Tukey HSD test. Effect of buck semen was evaluated by the comparison of the autumn data in both years using unpaired t test. The odds ratio among seasons was calculated from total values of cleavage and blastocyst rates in each season. Analyses were performed in BioEstat 5.3, with 95% CI. A value of *p* < 0.05 was considered as significant.

## 3. Results

**Figure 1:**
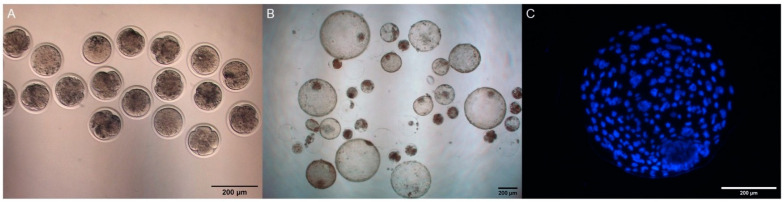
In vitro produced goat embryos. (**A**) Cleavage rate on Day 2 after IVF assessed by stereomicroscope; (**B**) Blastocysts on Day 8 after IVF assessed by stereomicroscope; (**C**) expanded blastocyst stained with Hoechst 33258 assessed by epifluorescence inverted microscope. The scale bar is set at 200 μm.

No differences (*p* > 0.05) were found in the autumn season between the two bucks used in the IVP outcomes (cleavage rate: 76 ± 1.9 vs. 66 ± 3.4%; blastocyst rate/cleaved: 75 ± 2.1 vs. 64 ± 4.4%; blastocyst rate from the initial number of COCs: 58 ± 2.6 vs. 42 ± 2.0%; and hatching rate: 70 ± 4.1 vs. 66 ± 4.1%). Thus, data were pooled regardless of the buck and analyzed together. Results of embryo development for different seasons of the year are presented in Table 1 and Figure 1. The cleavage rate was lower (*p* < 0.05) in spring, in comparison with either autumn or summer, while winter had intermediate values, being similar to all the others. Blastocyst formation rate from the initial number of COCs subjected to IVM was higher (*p* < 0.05) in autumn (52 ± 2.5%) as compared with other seasons (combined rates of the three seasons: 40 ± 1.9%). The hatching rate was similar (*p* > 0.05) among all seasons as well as the average number of cells in expanded blastocysts.

**Figure 2:**
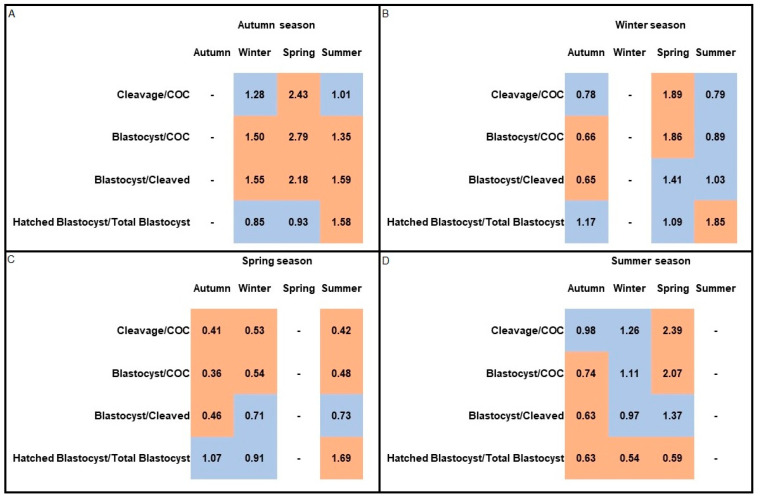
Heat map results of odds ratio (OR) of in vitro embryo production from adult goat oocytes in each season of the year for cleavage and blastocysts rates: (**A**) autumn season compared to all other seasons; (**B**) winter season compared to all other seasons; (**C**) spring season compared to all other seasons; and (**D**) summer season compared to all other seasons. The orange color indicates when the OR value was significant (*p* < 0.05), while the blue color represents OR values that were not significant (*p* > 0.05).

Regarding the odds ratio among seasons, the seasonality influence on in vitro embryo production was evident (Figure 2). Cleavage rate was negatively affected (*p* < 0.05) in spring in comparison to all the other seasons, with a greater chance of cleaving in the summer (OR: 2.39) and autumn (OR: 2.43). Similarly, the OR for the blastocyst rate from the initial number of COC was higher (*p* < 0.05) in autumn compared to the rest of seasons, mostly spring (OR: 2.79), and lower in the spring, compared to winter (OR: 0.54) and summer (OR: 0.48). There was a higher possibility to produce blastocysts from cleaved embryos during the autumn compared to the other seasons, mostly when compared to spring (OR: 2.18). The blastocysts had a lower (*p* < 0.05) probability to hatch in the summer.

## 4. Discussion

This study assessed the effects of reproductive seasonality on abattoir-derived oocyte developmental competence and overall IVP efficiency in adult goats over 1.5 years. Considering that caprine is a seasonal breeder species and that there is a great influence of seasons on their hormonal and systemic changes, it would be reasonable to hypothesize that the season could significantly affect the IVP outcomes. Two main conclusions may be drawn from our study. Firstly, a significant effect of the time of year was found, favoring the breeding season. Secondly, the blastocysts produced had a similar quality (cell number) throughout the years. Although it is well known that heat stress can affect the oocyte developmental competence [7], we believe this was not a confounding effect in our study, since winter and summer (most extreme temperatures) had similar and intermediate rates of embryo development. Of note, the season may reflect different strategies in reducing surplus animals due to farm management and, obviously, the number and status of slaughterhouse animals throughout the year are unknown. Despite this possible bias, the use of slaughterhouse ovaries is practically the only option available when aiming to use a great number of COCs and replicates. In the current study, the differences in the number of replicates across seasons is related to the overall laboratory logistics.

Since we had no difference throughout both years, we pooled data regardless of the years. Our data corroborate a previous study that reported similar rates of cleavage and blastocyst within each season over years in sheep [15]. This was expected since the breeding season is usually stable from year to year, with constant dates of onset and interruption of ovulatory activity (Reviewed by Chemineau, et al. [5]).

In the present study, cleavage rate was significantly lower in the anestrus (spring: 51%), in comparison with the breeding season (autumn: 72%) or transition (summer: 71%) while the winter (66%) had intermediate values. The effect of season on the cleavage rate in photoperiodic species is not unanimous in the literature. Cleavage was higher in the breeding season in the cat [12], plains bison [22], deer [23] and rhesus monkey [24]. Results in sheep varied regarding the effect of season on cleavage rate, since it was either affected [11,13] or not [15]. Overall, these data demonstrate it may be risky to consider cleavage will undoubtedly be affected by season.

In prepubertal goats, an opposite pattern in cleavage was observed, and an increase of 30% was reported in the anestrus, compared to breeding season in IVF-derived embryos (64 vs. 34%). Interestingly, the authors demonstrated that after parthenogenetic activation, cleavage rate was similar throughout the year [16]. The parthenogenetic activation is a great tool to assess the intrinsic competence of oocytes [17], bypassing the male factors. Therefore, it is reasonable to suggest that in such a case anestrus oocytes had a low ability to be fertilized, whereas meiotic maturation and developmental capacities were not affected. It is also important to consider that spermatozoa quality could have affected these results. Altogether, the results of the present study corroborate most literature on seasonal breeders and indicate that the fertilizing competence of the oocytes was probably affected in the anestrus season and/or the IVM-IVF conditions were not adapted to oocytes collected during the anestrus season. In addition, the developmental competence was also decreased in anestrus since both the rates of blastocysts calculated from total or cleaved oocytes were both lower in anestrus than in estrus.

The blastocyst rate from the initial number of COCs subjected to IVM was significantly higher in the breeding (autumn: 52%) as compared with other seasons (other three seasons: ⁓40%). This result was corroborated by the OR determined in breeding season, which was significantly higher than the other three seasons. These data are in agreement with a previous study in Sarda sheep, where higher blastocyst rate was achieved during the breeding season [15]. This rate may be considered as an indicator of the success of IVP in terms of embryo yield. The blastocyst rate from the cleaved embryos was higher in the breeding (autumn: 73%) compared to the anestrus season (spring: 55%; OR: 2.18). This parameter is of utmost importance concerning the cytoplasmic competence since it specifically gives information about those embryos that were able to overcome the genome activation stage [25], which occurs in the 8–16 cells stage in goats [26]. Conversely, once cleaved, embryos had similar developmental competence to reach the blastocyst stage in plain bison [22] and in prepubertal goats [16]. Monkey oocytes recovered during the breeding season had similar IVP rates to those recovered during anestrus, even though they had significantly higher developmental competence when the oocytes were subjected to FSH stimulation [24]. It is noteworthy that all seasons produced an adequate and even high rates of cleavage and blastocysts. Perhaps these facts justify the extremely low number of studies proposing alternatives to mitigate the season effect in goat IVP programs throughout the year. For instance, a melatonin implant seemed to improve sheep oocyte developmental competence during anestrus [27]. These data indicate that although oocytes may present lower competence, strategies could be applied in the anestrus season in order to alleviate the negative effects of season. The identification of molecular approaches for understanding and alleviating seasonal effects may open the way to more adapted methods [4] to maintain the same level of blastocyst production in the anestrus season.

Embryo quality was estimated based on the total number of blastomeres in expanded blastocysts on Day 8, which did not differ significantly among the four seasons. In the present study, the blastocysts had an average of 193 cells, higher than 120 [28], similar to 187 [17] and lower than 243 cells (but counted on Day 9) [29] of all studies in goats. Although the blastocyst morphological appearance is not definitely related to true developmental status in goats [28], all blastocysts allotted to the blastomeres count in the present study had an expected cell number (ranged from 168 to 225) compared to the literature [17,28,29]. Similarly, we found no significant differences between seasons in the number of hatched embryos. However, it was observed that blastocyst hatching was less probable during summer compared to the other seasons. Indeed, it is well known that even though the IVP success in terms of blastocyst production depends on oocyte intrinsic quality and IVM conditions, the quality of the subsequent blastocysts relies on the IVD system [30] and the IVD system remained the same throughout experiments. These data suggest that it is possible to produce goat embryos of similar quality throughout the years, regardless of the season.

## 5. Conclusions

Results of the present study indicate that the season has a significant impact on the IVP outcomes of adult goats. The breeding (autumn) season leads to improved oocyte developmental competence, resulting in higher cleavage and blastocyst yield, while there is no difference in embryo quality throughout the years. Strategies to mitigate the season effect in the anestrus (spring) season must be proposed to enhance the repeatability of the results throughout the year in goat IVP programs.

## Figures and Tables

**Table 1:** Effect of different seasons on developmental competence of adult goat oocytes derived from slaughterhouse ovaries. Percentages of cleavage, blastocysts (Bl), and hatched embryos in relation to the total blastocysts (Hbl/totBl), and blastocyst cell counts (Mean ± S.E.M.).

Season	*n*	Replicates	Cleavage (%)	Bl/COC (%)	Bl/Cleaved (%)	Hbl/totBl (%)	Total Cells
Autumn	811	17	72 ± 2.1 ^a^	52 ± 2.5 ^a^	73 ± 2.7 ^a^	68 ± 2.9	198 ± 4.6
Spring	404	7	51 ± 7.1 ^b^	28 ± 4.7 ^b^	55 ± 2.6 ^b^	65 ± 3.8	187 ± 3.6
Summer	639	15	71 ± 2.0 ^a^	45 ± 2.3 ^c^	63 ± 3.3 ^a,b^	76 ± 5.1	191 ± 3.3
Winter	494	10	66 ± 4.1 ^a,b^	42 ± 2.1 ^c^	63 ± 4.1 ^a,b^	67 ± 4.4	196 ± 4.2
Total	2348	49	67 ± 1.8	44 ± 1.7	65 ± 1.8	66 ± 2.0	193 ± 2.0

*n*: Number of oocytes submitted to in vitro fertilization and development. a,b,c: Within a column values with different superscripts differ significantly (*p* < 0.05).

## Data Availability

The data presented in this study are available on request from the corresponding author.
